# Clinical, genetic, and cognitive correlates of seizure occurrences in Phelan-McDermid syndrome

**DOI:** 10.1186/s11689-024-09541-0

**Published:** 2024-05-10

**Authors:** Tess Levy, Jacob Gluckman, Paige M. Siper, Danielle Halpern, Jessica Zweifach, Rajna Filip-Dhima, J. Lloyd Holder, M. Pilar Trelles, Kristina Johnson, Jonathan A. Bernstein, Elizabeth Berry-Kravis, Craig M. Powell, Latha Valluripalli Soorya, Audrey Thurm, Joseph D. Buxbaum, Mustafa Sahin, Alexander Kolevzon, Siddharth Srivastava, on behalf of the Developmental Synaptopathies Consortium

**Affiliations:** 1 Seaver Autism Center for Research and Treatment, Icahn School of Medicine at Mount Sinai, New York, NY 10029 USA; 2 Department of Psychiatry, Icahn School of Medicine at Mount Sinai, New York, NY 10029 USA; 3 The Mindich Child Health and Development Institute, Icahn School of Medicine at Mount Sinai, New York, NY 10029 USA; 4 Department of Neurology, Rosamund Stone Zander Translational Neuroscience Center, Boston Children’s Hospital, Harvard Medical School, 300 Longwood Avenue, Boston, MA 02115 USA; 5 F.M. Kirby Neurobiology Center, Boston Children’s Hospital, Harvard Medical School, Boston, MA 02115 USA; 6 Department of Pediatrics, Division of Neurology and Developmental Neuroscience, Baylor College of Medicine, Houston, TX 77030 USA; 7 Jan and Dan Duncan Neurological Research Institute, Texas Children’s Hospital, Houston, TX 77030 USA; 8 Department of Electrical & Computer Engineering, Northeastern University, Boston, MA 02115 USA; 9 Department of Communication Sciences & Disorders, Northeastern University, Boston, MA 02115 USA; 10 Department of Pediatrics, Stanford University School of Medicine, Stanford, CA 94304 USA; 11 Department of Pediatrics, Rush University Medical Center, Chicago, IL 60612 USA; 12 Department of Neurological Sciences, Rush University Medical Center, Chicago, IL 60612 USA; 13 Department of Anatomy and Cell Biology, Rush University Medical Center, Chicago, IL 60612 USA; 14 Department of Neurobiology, University of Alabama at Birmingham Heersink School of Medicine, Birmingham, AL 35233 USA; 15 Civitan International Research Center, University of Alabama at Birmingham, Birmingham, AL 352233 USA; 16 Department of Psychiatry & Behavioral Sciences, Rush University Medical Center, Chicago, IL 60612 USA; 17 Neurodevelopmental and Behavioral Phenotyping Service, National Institute of Mental Health, National Institutes of Health, Bethesda, MD 20892 USA; 18 Friedman Brain Institute, Icahn School of Medicine at Mount Sinai, New York, NY 10029 USA; 19 Department of Genetics and Genomic Sciences, Icahn School of Medicine at Mount Sinai, New York, NY 10029 USA; 20 Department of Neuroscience, Icahn School of Medicine at Mount Sinai, New York, NY 10029 USA; 21 Department of Pediatrics, Icahn School of Medicine at Mount Sinai, New York, NY 10029 USA

**Keywords:** Phelan-McDermid syndrome, 22q13, SHANK3, Seizures, Epilepsy

## Abstract

**Background:**

Phelan-McDermid syndrome (PMS) is a genetic neurodevelopmental disorder caused by *SHANK3* haploinsufficiency and is associated with an increased risk for seizures. Previous literature indicates that around one third of individuals with PMS also have epilepsy or seizures, with a wide range of types and ages of onset. Investigating the impact of seizures on intellectual and adaptive functioning for PMS is a primary concern for caregivers and is important to understanding the natural history of this syndrome.

**Methods:**

We report on results from 98 individuals enrolled in a prospective, longitudinal study. We detailed seizure frequency, type, and age of onset, and we analyzed seizure occurrence with best estimate IQ, adaptive functioning, clinical features, and genotype. We conducted multiple linear regression analyses to assess the relationship between the presence of seizures and the Vineland Adaptive Behavior Scale, Second Edition (VABS-II) Adaptive Behavior Composite score and the best estimate full-scale IQ. We also performed Chi-square tests to explore associations between seizure prevalence and genetic groupings. Finally, we performed Chi-square tests and t-tests to explore the relationship between seizures and demographic features, features that manifest in infancy, and medical features.

**Results:**

Seizures were present in 41% of the cohort, and age of onset was widely variable. The presence of seizures was associated with significantly lower adaptive and intellectual functioning. Genotype–phenotype analyses were discrepant, with no differences in seizure prevalence across genetic classes, but with more genes included in deletions of participants with 22q13 deletions and seizures compared to those with 22q13 deletions and no seizures. No clinical associations were found between the presence of seizures and sex, history of pre- or neonatal complications, early infancy, or medical features. In this cohort, generalized seizures were associated with developmental regression, which is a top concern for PMS caregivers.

**Conclusions:**

These results begin to eludicate correlates of seizures in individuals with PMS and highlight the importance of early seizure management. Importantly, presence of seizures was associated with adaptive and cognitive functioning. A larger cohort might be able to identify additional associations with medical features. Genetic findings suggest an increased capability to realize genotype–phenotype relationships when deletion size is taken into account.

**Supplementary Information:**

The online version contains supplementary material available at 10.1186/s11689-024-09541-0.

## Background

Phelan-McDermid syndrome (PMS) is a genetic disorder caused by haploinsufficiency of *SHANK3*, either by 22q13 deletion or pathogenic sequence variant. In addition to seizures, affected individuals present with a wide spectrum of systemic abnormalities and neurodevelopmental challenges, including autism spectrum disorder (ASD), intellectual disability (ID, often severe to profound), behavioral problems, gastrointestinal problems, and other medical features including renal and cardiac abnormalities [1–4].

Seizures are among the most complex comorbidities to manage and represent a main concern of caregivers [5–7]. The pooled prevalence of seizures based on prior literature is 32% [8]. Individual studies have a prevalence ranging from 17 to 70% (see [8]). Reported seizure types include atypical absence, tonic, atonic, tonic–clonic, and myoclonic seizures. Seizure frequency spans a wide range, from a single lifetime seizure to intractable epilepsy with hundreds of daily seizures [8–10]. Many abnormal electroencephalogram (EEG) findings are reported in individuals with PMS and seizures, including slowing or absence of the occipital dominant rhythm and multifocal paroxysmal abnormalities [8, 9]. Abnormal EEG findings have also been reported in PMS individuals without seizures, including slow occipital dominant rhythm or focal spike and slow wave activity [8, 9].

There is limited information about factors which predict prevalence or severity of seizures in PMS. A recent large genotype–phenotype study in PMS has suggested that there is no statistically significant difference in prevalence of epilepsy when comparing those with sequence variants and Class 1 deletions (deletions including only *SHANK3* or *SHANK3* in combination with *ARSA* and/or *ACR* and *RABL2B*) versus those with Class 2 deletions (all other deletions). The prevalence of epilepsy was 26% (19/73) in the former and 27% (22/83) in the latter [11]. Additional literature has found no statistically significant differences between seizure prevalence in individuals with sequence variants (7/10) as compared to those with deletions (15/36) but did identify that deletion size was larger in individuals with seizures compared to those without seizures [10].

Furthermore, it is unclear whether other clinical features of PMS are related to presence of seizures. Investigating if seizures affect intellectual and adaptive functioning, for example, is important to understanding the natural history of this syndrome and represents a key caregiver concern [5]. Identifying associations with clinical features in infancy or early childhood may also predict who may be at a greater risk of developing seizures.

Here, we present the findings from a natural history study in PMS, focusing this analysis on seizure characteristics. We hypothesize that the presence of seizures is associated with lower cognitive and adaptive functioning. We performed additional exploratory analyses comparing genetic and clinical features to seizure occurrence.

## Methods

### Study participants

We performed analysis of longitudinal data collected from a prospective, multi-site, observational study evaluating the phenotype and natural history of PMS (ClinicalTrials.gov NCT02461420). Eligibility criteria included presence of a chromosomal 22q13 deletion including *SHANK3* or a pathogenic *SHANK3* sequence variant; ability to understand English; age 3–21 years at time of enrollment. All *SHANK3* variants were classified as likely pathogenic or pathogenic according to ACMG-AMP criteria [12], all were de novo when both parental samples were available for testing. Genetic information including breakpoints for deletions and variant information are provided in Supplemental Table 1. Diagnostic and Statistical Manual, 5th Edition (DSM-5) ASD diagnoses were made upon review of Autism Diagnostic Observation Schedule, 2nd Edition and/or Autism Diagnostic Interview – Revised, along with a clinical evaluation [13–15].

### Seizure characteristics

Seizure history was collected through a clinical interview with caregivers with review of medical records when available. Interval histories were collected at each follow up timepoint. Caregivers were asked if their child had *any* seizure and if so, if they had febrile seizures. They were also asked about a formal epilepsy diagnosis. A separate interview form assessed seizure characteristics where caregivers were asked about the type and subtype of seizures, as well as onset date. Caregiver report was based on prior clinical assessment of seizures and medical records of these assessments were provided when available. This study did not involve clinical EEGs, MRIs, or other seizure assessments. Seizure types were organized according to the International League Against Epilepsy guidelines: focal (including with impairment of consciousness, without impairment of consciousness, evolving to secondary generalization); generalized (including motor subtypes: tonic–clonic, myoclonic, clonic, tonic, atonic, and epileptic spasms and nonmotor (absence seizures) [16]; and seizure not classifiable as focal or generalized. If seizure type was not reported, it was considered unclassified. Prevalence of seizure is defined as occurrence at any time point in the study; however, when conducting linear regressions with baseline adaptive and cognitive measures (see below), we used seizure prevalence at the baseline timepoint in the study. We did not differentiate between provoked and unprovoked seizure or between seizure in the setting of an epilepsy diagnosis vs. seizure in absence of epilepsy diagnosis. We did not quantify seizure frequency but rather whether the seizure type was present or not. We defined having any seizures as having one or more of the following seizure types: focal, generalized, unclassified epileptic event; this included febrile seizures. Date of seizure onset was collected whenever possible; age of seizure onset was calculated from onset of seizure and date of birth. Supplemental Tables 2 and 3 display seizure proportions and age of onset in participants with a reported epilepsy diagnosis.

### Analysis of seizure prevalence with adaptive functioning and cognitive ability

Multiple linear regression analyses assessed the relationship between the presence of seizures at the time of baseline assessment and the baseline Vineland Adaptive Behavior Scale, Second Edition (VABS-II) Adaptive Behavior Composite standard score, as well as the baseline best estimate full-scale IQ. The composite score is a standard score with a general population mean of 100 and standard deviation of 15. Best estimate IQ was generated, which combines standard IQ scores for those who were in age range of cognitive assessment and ratio IQ estimates for those who were out of range for cognitive tests and/or who performed at the floor of IQ testing; tests included the Stanford-Binet Intelligence Scales, 5th Edition, the Differential Abilities Scales, 2^nd^ Edition, and the Mullen Scales of Early Learning [17, 18, 19]. VABS-II scores and best estimate IQ scores were used from the baseline timepoint. Covariates were added to the model, including age, ASD diagnosis, and genetic group (Class 1 deletions, Class 2 deletions, sequence variants). The analyses were done with baseline seizure status and baseline VABS-II and full scale IQ (FSIQ). Additional covariates including sex, age of onset of seizures, and family history of seizures were not significant and did not remain in the model. Separate models assessed the relationship of 1) any seizures 2) generalized seizures and 3) focal seizures.

### Genotype–phenotype associations

Chi-square tests explored if there were differences between seizure prevalence at any timepoint in the study and genetic groupings (class 1 deletions, class 2 deletions, sequence variants). Class 1 deletions are 22q13 deletions that include only *SHANK3* with or without the deletion of *ARSA*, *ACR*, and *RABL2B*. These latter three genes are not expected to contribute to the phenotype of PMS because they are not constrained for protein truncating variants (pLI = 0 in gnomAD database). *ARSA* is associated with a known autosomal recessive disorder. Class 2 deletions are larger deletions that do not qualify as Class 1 deletions (i.e., including the deletion of any other genes in addition to *SHANK3* and the three mentioned above). Sequence variants are pathogenic sequence variants within the *SHANK3* gene. For participants with deletions, the number of genes included in the deletion was evaluated using hg19 coordinates. Wilcoxon tests explored if the presence of any seizures (focal, generalized, unclassified) and seizure types (focal, generalized) were associated with the number of genes deleted. This analysis was able to explore the relationship with the size of deletion rather than grouping all deletions that extend past *ARSA* into one group. Lastly, we described prevalence of seizures in participants with PMS who have a ring chromosme 22.

### Clinical associations

Chi square tests and t-tests explored if the presence of any seizures at any timepoint (focal, generalized, unclassified) were associated with demographic features, early infancy features, and medical features. Additional exploratory chi square tests examined if developmental regression was associated with any seizure, generalized seizures, or focal seizures.

Developmental regression was measured by the Autism Diagnostic Interview, Regression Supplement and by caregiver report during the clinical exam at each timepoint. Regression was defined by the loss of skills previously obtained for at least 3 months. Skills captured in the Regression Supplement are early skills (e.g., crawling, pointing, walking, babbling). Loss at any age was included in analyses; analyses did not differentiate between the two peaks of regression in PMS (childhood, adolescent). Age of skill loss was recorded for the skill regressions. Chi square tests assessed the relationship between history of skill regression and the presence of any seizures, generalized seizures, and focal seizures. For participants with both seizure and skill loss history, ages of each were compared to assess if skill loss came before or after seizures.

### Medication and seizure management

Anti-seizure medication (ASM) use was collected through caregiver survey. Changes to medications were reported through interval history forms during follow-up visits. Similarly, surgical history was collected through caregiver survey at baseline and each follow-up visit.

## Results

### Study participants

Ninety-eight participants were included in analyses and 45/98 (46%) were female. Average age at time of enrollment was 8.8 (4.6) years, with a range of 3–21 years. At baseline, mean VABS-II composite score was 51.2 (13.9), and best estimate IQ was 26.2 (17.8). There were 26 participants with Class 1 deletions, 53 with Class 2 deletions, and 19 with sequence variants.

### Seizure characteristics

Through the course of the study, 42% (41/98) of the cohort reported a history of any seizure type, including generalized seizures (27%, 26/98); focal seizures (20%, 20/98), or unclassified seizure type (13%, 13/98) (Table 1). Two participants reported febrile seizures only, which were included in any seizure type and unclassified seizure type. Prevalence of specific generalized and focal seizure subtypes are shown in Table 1. Caregivers of 19 participants reported a formal epilepsy diagnosis; seizure subtypes for participants with a reported epilepsy diagnosis are located in Supplemental Tables 2 and 3.

**Table 1:** Seizure types ever experience by participants in the cohort

Seizure type		Count	Overall proportion	Proportion of those with seizures
Any seizure		41/98	42%	100%
Epilepsy diagnosis		19/98	19%	46%
Febrile seizure		18/98	18%	44%
Generalized seizure		26/98	27%	63%
Motor subtypes	Tonic–Clonic	13/98	13%	32%
	Myoclonic	2/98	2%	5%
	Clonic	3/98	3%	7%
	Tonic	5/98	5%	12%
	Atonic	3/98	3%	7%
	Epileptic Spasms	1/98	1%	2%
Nonmotor	Absence	14/98	14%	34%
Focal seizure		20/98	20%	49%
	Without Impairment of Consciousness	9/98	9%	22%
	With Impairment of Consciousness	11/98	11%	27%
	Evolving to Bilateral Convulsive	3/98	3%	7%
Unclassified seizure		13/98	13%	32%

*Legend*: Seizure counts and prevalence in the cohort. Prevalence is listed as overall prevalence (entire cohort) and prevalence within those who have seizures (i.e., out of 41). Participants may have had multiple seizure types so percentages do not add up to totals

**Fig. 1:**
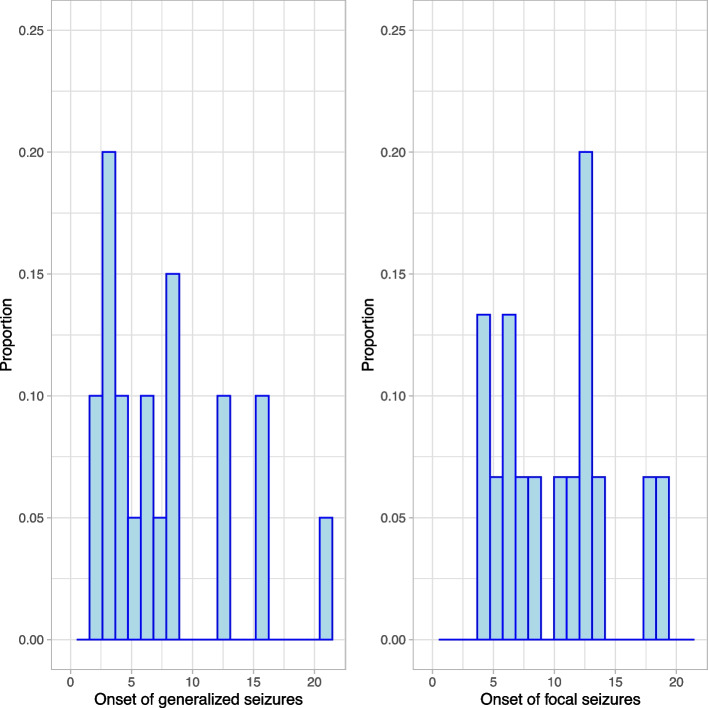
Seizure onset. *Legend*: Histogram of the age of seizure onset (years). The Y axis is the proportion of individuals. Bin width is 1 year

There were 12 participants with generalized seizures only, five with focal seizures only, and eight with unclassified seizures only; 13 participants had both generalized and focal seizures. Two participants had generalized, focal, and unclassified seizures reported, and one participant had generalized and unspecified seizures reported. True count and prevalence of generalized and focal seizures may be different as unspecified seizures can be either focal or generalized. For participants who reported the date of seizure onset, average age of onset of each type of seizure is displayed in Table 2 and Fig. 1.

**Table 2:** Age of onset of seizures for those with this data available

Seizure type		Age of onset (Years) (Mean, SD)	N available
Generalized (earliest)		7.75 (5.3)	20/26
Motor subtypes	Tonic–Clonic	9.74 (6.3)	11/13
	Myoclonic	8.61 (7.0)	2/2
	Clonic	10.72 (6.2)	3/3
	Tonic	5.96 (2.7)	4/5
	Atonic	8.20 (0.9)	2/3
	Epileptic Spasm	3.73 (.)	1/1
Nonmotor	Absence	7.56 (4.5)	9/14
Focal (earliest)		10.35 (4.7)	15/20
	Without Impairment of Consciousness	7.78 (3.5)	3/9
	With Impairment of Consciousness	9.8 (5.1)	9/11
	Evolving to Bilateral Convulsive	9.60 (4.3)	3/3

*Legend*: Mean and standard deviation of age of seizure onset (years). The number of participants with available dates to calculate onset age are in the last column. Participants may have had multiple seizure types so subgroup sample size does not add up to overall totals

### Association of seizure prevalence with adaptive functioning and cognitive ability

**Fig. 2:**
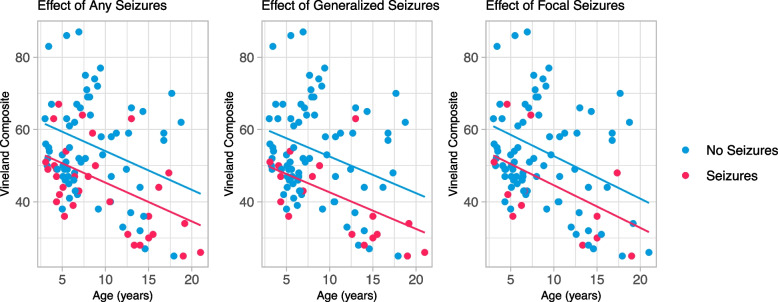
Effect of seizures on Vineland Adaptive Behavior Composite. *Legend*: Multiple linear regression including presence of seizures at baseline with VABS-II Adaptive Behavior Composite. Blue scatterpoints and line represent individuals without seizures (left), without generalized seizures (middle), or without focal seizures (right). Red scatterpoints and line represent individuals with any seizures (left), generalized seizures (middle), or focal seizures (right). The X axis is age in years. Downward slope indicates that skills are not increasing at a rate comparable with the general population not that skills are declining

At baseline, presence of any seizures, generalized seizures, and focal seizures were all significant predictors of the VABS-II composite standard score. The composite score was 8.73 [-13.3- -4.2] points lower in individuals with any seizures compared to those without seizures, while controlling for age, ASD diagnosis, and genetic grouping (*p* = 0.0003) (Fig. 2). The presence of focal seizures had a similar impact, though with more variability, with a composite score 8.07 [-14.8- -1.4] points lower in those with focal seizures, while controlling for age, ASD diagnosis, and genetic grouping (*p* = 0.019). Generalized seizures had a slightly greater impact on the composite score, where those with generalized seizures had a score 9.90 [-15.6- -4.2] points lower than individuals without generalized seizures, while controlling for the same covariates (*p* = 0.0008). Bonferonni corrections for multiple comparisons would provide an alpha of 0.017, leaving the categories of any seizures and generalized seizures remaining statistically significant and the category of focal seizures just above the threshold for statistical significance.

**Fig. 3:**
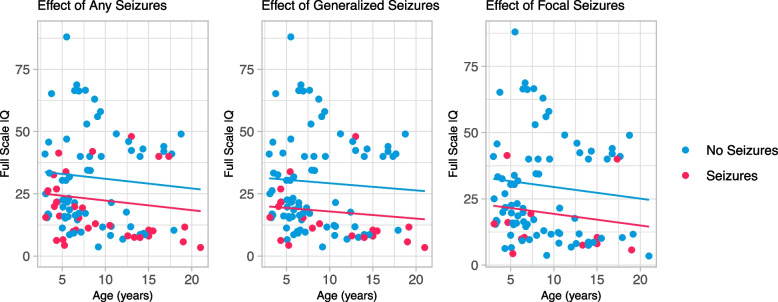
Association between occurrence of seizures and best estimate full scale IQ. *Legend*: Multiple linear regression of seizure occurrence at baseline and the best estimate full scale IQ. Blue scatterpoints and line represent individuals without any seizures (left), generalized seizures (middle), or focal seizures (right). Red scatterpoints and line represent individuals with seizures (left), generalized seizures (middle), or focal seizures (right). The X axis is age in years. Downward slope indicates that skills are not increasing at a rate comparable with the general population not that skills are declining

Similarly, presence of any seizures, generalized seizures, and focal seizures at baseline were all significant predictors of best estimate full-scale IQ. Best estimate FSIQ was 8.74 [-14.6- -2.9] points lower in individuals with any seizures compared to those without seizures, while controlling for age, ASD diagnosis, and genetic grouping (*p* = 0.004) (Fig. 3). Best estimate FSIQ was 10.14 [-18.6- -1.7] points lower in those with focal seizures compared to individuals without focal seizures, while controlling for age, ASD diagnosis, and genetic grouping (*p* = 0.025), again showing wider variability. Generalized seizures had the greatest impact on FSIQ where those with seizures had a score 11.33 [-18.5- -4.13] points lower than individuals without generalized seizures, while controlling for the same covariates (*p* = 0.002). Bonferonni corrections for multiple comparisons would provide an alpha of 0.017, leaving any seizure and generalized seizures significant and focal seizures just over the significance value.

When assessing cognitive and adaptive scores within participants with seizures across either one (generalized or focal) or two (generalized and focal) seizure types at baseline, participants with two seizure types tended to have lower, but not significantly lower, scores, compared to participants with only one seizure type. Using the same model with age, genetics, and ASD held constant, participants with both generalized and focal seizures at baseline had an Adaptive Behavior Composite score 5.30 points lower [-14.7 – 4.1] (*p* = 0.25), and an FSIQ points 7.79 lower [-18.3 – 2.7] (*p* = 0.14) than individuals with only focal or generalized seizures.

### Genotype–phenotype associations

No differences were found between genetic subgroups and the presence of seizures or any specific type of seizure at any timepoint. Eight of 26 (31%) subjects with a Class 1 deletion, 26/53 (49%) of subjects with a Class 2 deletion, and 7/19 (37%) with a sequence variant reported any seizure type (*p* = 0.27). 3/26 (12%) with a Class 1 deletion, 17/53 (32%) with a Class 2 deletion, and 6/19 (32%) with a sequence variant reported generalized seizures (*p* = 0.13). Five of 26 (19%), 11/53 (21%), and 4/19 (21%) of the Class 1, Class 2, and sequence variant group reported focal seizures, respectively (*p* = 0.98).

**Fig. 4:**
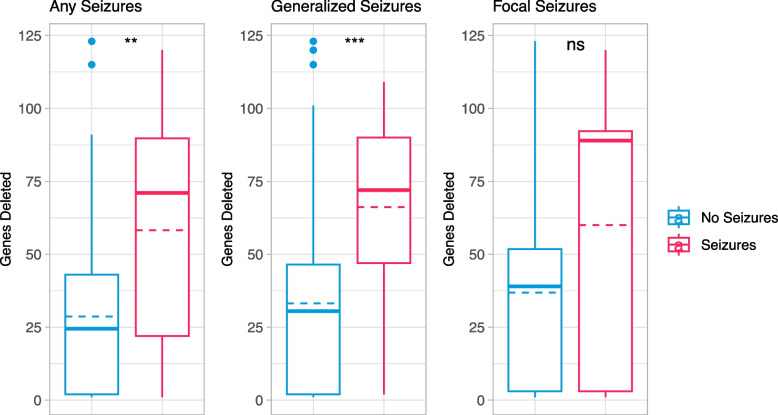
Number of genes included in deletions of participants with and without seizures. *Legend*: Boxplots representing the number of genes deleted in participants’ deletions for those with chromosomal deletions and without seizures (blue) or with seizures (red). The minimum and maximum values are represented with tails, the box represents the interquartile range, the bold line represents the median, and the dashed line represents the mean genes deleted per group. Asterisks represent significance level, ** *p* <  = 0.01, *** *p* <  = 0.001, ns *p* > 0.05

Among participants with chromosomal deletions (*n* = 79), those who had any seizure type had a median of 71 (IQR: 68) genes deleted as compared to individuals without seizures who had a median of 25 (41) genes deleted (*p* = 0.002) (Fig. 4). Participants with chromosomal deletions and generalized seizures had median of 72 (43) genes deleted as compared 31 (45) for those without generalized seizures (*p* = 0.0005). No significant differences were found in the presence of focal seizures, where subjects with chromosomal deletions and seizures had a median of 89 (89) genes deleted compared to 39 (49) in individuals without focal seizures. The differences in any seizures and generalized seizures were significant after Bonferonni corrections, with an alpha of 0.017.

Individuals with PMS who have a ring chromosome had a similar seizure prevalence (2/6, 33%) compared to the rest of the cohort. These two participants both had focal and generalized seizures.

### Clinical associations

The presence of any seizures at any timepoint was not associated with any demographic, pre- or neonatal complications, early infancy features, or medical features (Table 3).

**Table 3:** Prevalence of clinical features in participants with and without seizures

Domain	Feature	Without seizures	With seizures	*P* value
Demographics	Sex (Female)	27/57 (47%)	18/41 (44%)	0.89
	Age	11 (4.1)	11 (5.3)	0.96
	Ethnicity	4/56 (7%)	7/39 (18%)	0.20
Pre- and neonatal	Pregnancy complications	23/55 (42%)	14/39 (36%)	0.72
	Preterm birth	15/53 (28%)	5/41 (12%)	0.10
	Need for NICU stay	14/55 (25%)	14/40 (35%)	0.44
Early infancy features	Sleep disruption	20/52 (38%)	22/39 (56%)	0.137
	Irritability	17/53 (32%)	10/39 (26%)	0.66
	Decreased alertness	14/52 (27%)	15/37 (41%)	0.26
	Lethargy	11/50 (22%)	10/39 (26%)	0.88
	Hypotonia	33/51 (65%)	29/39 (74%)	0.45
Medical features	Head trauma	7/57 (12%)	9/40 (23%)	0.29
	Cardiac defects	8/56 (14%)	8/38 (21%)	0.56
	Renal abnormalities	9/51 (18%)	11/34 (32%)	0.19
	Recurrent infections	15/56 (27%)	11/38 (29%)	1
	Sleep disturbance	27/55 (49%)	19/38 (50%)	1
	Microcephaly	3/49 (6%)	4/38 (11%)	0.73
	Macrocephaly	4/51 (8%)	5/38 (13%)	0.64

Developmental regression was not significantly associated with any seizure type or focal seizures at any timepoint, but was associated with generalized seizures (*p* = 0.003). Of the 26 subjects with generalized seizures, 18 (69%) of them had a reported developmental regression. Of the 72 participants without generalized seizures, 24 (33%) had a reported regression. For participants who reported both age of first seizure and age of regression, 13/16 (81%) had a reported regression prior to a seizure while the remaining 3/16 (19%) had a reported regression after a first seizure.

### Medication and seizure management

Participants used multiple different ASMs. The three most commonly used ASMs were levetiracetam, valproic acid, and lamotrigine (Table 4). Of those who ever used ASMs (*n* = 25), over half (*n* = 15) used only one ASM, while 10 participants used two or more ASMs, and one participant used 7 different ASMs. Use of as needed diazepam and midazolam were not included in ASM counts. One participant had a corpus callosotomy for refractory epilepsy, five years later the patient had a placement of a vagus nerve stimulator, which was replaced four years after that.

**Table 4:** Anti-seizure medications utilization

Medication	Count
Levetiracetam	14
Lamotrigine	6
Valproic acid	6
Diazepam^a^	5
Clobazam	4
Oxcarbazepine	4
Rufinamide	3
Topiramate	3
Clonazepam	1
ethosuximide	1
Felbamate	1
Midazolam^a^	1
Perampanel	1

*Legend*: Anti-seizure medication use in the cohort

^a^Could not ascertain if medications were used for maintenance or seizure rescue. All other medications were used for seizure maintenance

## Discussion

The prevalence of seizures in this cohort (42%) was similar, though slightly higher, than the previously reported pooled prevalence of 35% [8]. The frequency of seizure types is also representative of past literature, where absence seizures are the most common single finding, reported in 14 of the 41 participants with seizures (34%), followed by generalized tonic–clonic seizures which were reported in 13 of 41 (32%) of participants with seizures. This varies from the general population where focal seizures are considered more common in both children and adults [20], but is similar to some other neurodevelopmental disorders where generalized seizures are more frequent, such as Angelman syndrome [21].

Analyses of the age of onset of seizures showed no definitive point at which the risk for development of seizures comes to an end. On average, the onset of generalized seizures was 2.6 years earlier than the average onset of focal seizures. For individuals with generalized seizures, 75% had onset prior to 10 years old. The remaining 25% had onset that was largely variable, occurring through early adulthood. The risk for new onset generalized seizures does appear to lessen as individuals get older. For those with focal seizures, 47% had onset prior to 10 years old, an additional 40% of participants had onset between 10 and 15 years old, and the remaining subjects had onset in adulthood. This result is limited by the amount of data available for onset age but may indicate a difference in the pattern of onset between generalized and focal seizures in PMS. Additionally, this study only included participants who were 3–21 years old at time of enrollment, so seizure onset later than these ages was not captured.

Analyses of adaptive and intellectual functioning indicated that the presence of seizures was associated with lower scores. Individuals with seizures are estimated to have scores over half a standard deviation lower than individuals without seizures, after accounting for covariates including age and genotype. This finding was consistent across seizure types, with generalized seizures appearing to have the greatest magnitude of impact with intellectual and adaptive functioning. Results from focal seizure analyses were just above the alpha level using Bonferroni correction methods. As the onset of generalized seizures tended to be younger, the age of onset of seizures was explored as predictor of adaptive and intellectual scores, however, was not statistically significant. This may be due to the limited information on onset age and/or may indicate more complex etiology for the effect of generalized seizures and intellectual functioning. Literature in the general epilepsy population has shown negative relationships with cognitive functioning, with similar impairment due to focal and generalized seizures, and has suggested that younger onset of seizures is associated with more impairment [22, 23].

Identifying early onset clinical features associated with seizures may help shed light on which individuals with PMS may be at a greater risk of seizure development. Congenital features, such as cardiac and renal anomalies or complications such as preterm birth were not associated with seizures in this cohort. Additionally, infancy traits such as lethargy or decreased alertness were not associated with seizures. Features that reflect differences in brain morphology and growth such as microcephaly or macrocephaly also did not show associations with seizures in this cohort. Larger cohorts may be able to identify possible significant clinical associations. Additionally, older age was not associated with increased seizure prevalence in this cohort, unlike previously reported cohorts [1].

Type of genetic variation is another potential strategy to personalize seizure risk estimates. However, similar to past literature, results are not clear. There was no difference in the prevalence of seizures across participants with Class 1 (smaller), Class 2 (larger) deletions or *SHANK3* sequence variants, indicating that loss of *SHANK3* is a key factor in the etiology of seizures in PMS. However, when analyzing the number of genes deleted per participant against seizure prevalence, results showed individuals with deletions including more genes had a higher seizure rate, and specifically a higher generalized seizure rate. This implies that genes in the 22q13 region other than *SHANK3* may be implicated in the risk for generalized seizures. Clustering participants into genetic groups can aid in the interpretation of results, specifically to assess differences between sequence variants and Class 1 deletions (only *SHANK3* involvement) to Class 2 deletions (*SHANK3* plus other genes) but may miss differences within those classified as Class 2.

Developmental regression is a top concern for PMS caregivers, and to date it remains unclear why some individuals with PMS experience regression. Previous literature in PMS is conflicting as to whether regression and presence of seizures are related. In this cohort, generalized, but not focal, seizures were associated with regression, again pointing to a difference in severity of phenotype for those with generalized seizures. Upon review of the ages of seizures and regression, it appears most participants experienced a developmental regression *prior* to the onset of seizures. Though directionality cannot be assessed, these results do not suggest that seizures are a risk factor to a future regression.

Sixty-one percent of participants with seizures reported taking at least one ASM. The most common were levetiracetam, valproic acid, and lamotrigine. No specific recommendations exist for seizure management within PMS, other than standard epilepsy practice [6, 7]. Results show that over half (60%) of individuals with PMS and epilepsy who require medication have only ever used 1 ASM, which suggests seizures have been well controlled.

## Conclusions

Seizures are a common feature of PMS that carry a heavy burden for individuals and their caregivers. Results from this study indicate that seizures are associated with lower intellectual and adaptive functioning. Although seizure management should always be optimized, it is not clear there is a causative relationship between seizures and lower cognitive functioning, and the seizures may just reflect a pre-existing more severe synaptopathy which also drives the higher level of impairment. This concept is supported by the relationship between seizures and deletion of a larger number of genes, and that seizures did not often precede regression. Seizures are associated with premature mortality in the general population [24, 25] and cases of premature mortality in individuals with PMS have been reported, again underscoring the importance of targeted research and therapy development. There are no approved therapies targeting seizures in individuals with PMS yet, though clinical trials are underway. This study was limited by selection bias, where those with medical features such as epilepsy are more likely to be offered genetic testing, and therefore diagnosed with PMS, potentially overinflating the true prevalence of seizures in this disorder. The sample size was robust for a rare disease study but may be underpowered to identify clinical correlates of seizures. Lastly, we did not review EEG data, differentiate between unprovoked and provoked seizures, or differentiate between seizures in the setting of an epilepsy diagnosis (2 + unprovoked seizures or 1 unprovoked seizure with high chance of future seizures) vs. seizures generically (unprovoked, provoked, single lifetime seizure, etc.). Seizure types and epilepsy diagnoses were obtained from caregiver report and medical records when available, rather than prospective EEGs, representing another limitation of this study. Future studies with larger cohorts should re-evaluate early onset features of disease manifesting early in life that may pre-date seizure onset in PMS to help identify those at greater risk.

### Supplementary Information

Supplementary Material 1.

Supplementary Material 2.

Supplementary Material 3.

## Data Availability

Clinical data presented here have been deposited in the National Database for Autism Research (NDAR), an NIH-funded data repository that stores and shares data pertaining to Autism Spectrum Disorder with qualified researchers.

## Abbreviations

ASD: Autism Spectrum Disorder
ASM: Anti-Seizure Medication
DSM-5: Diagnostic and Statistical Manual, 5th Edition
EEG: Electroencephalogram
FSIQ: Full Scale IQ
gnomAD: Genome Aggregation Database
ID: Intellectual Disability
pLI: Probability of Loss-of-Function Intolerance
PMS: Phelan-Mcdermid Syndrome
VABS-II: Vineland Adaptive Behavior Scale, Second Edition
