# Supplementary material for: Clinical, genetic, and cognitive correlates of seizure occurrences in Phelan-McDermid syndrome
Source: J Neurodev Disord. 2024 May 10;16:25. doi: 10.1186/s11689-024-09541-0 (PMC11084001; doi:10.1186/s11689-024-09541-0)
Supplement: Supplementary file 2 — Supplementary Material 2. [file 11689_2024_9541_MOESM2_ESM.docx]

**Supplemental Table 2**. Seizure types ever experience by participants in the cohort with an epilepsy diagnosis.

| Seizure Type |  | Count | Proportion |
| --- | --- | --- | --- |
| Epilepsy Diagnosis |  | 19/19 | 100% |
| Febrile Seizure |  | 6/19 | 32% |
| Generalized Seizure |  | 12/19 | 63% |
| Motor subtypes | Tonic-Clonic | 8/19 | 42% |
|  | Myoclonic | 0/19 | 0% |
|  | Clonic | 2/19 | 11% |
|  | Tonic | 4/19 | 21% |
|  | Atonic | 2/19 | 11% |
|  | Epileptic Spasms | 1/19 | 5% |
| Nonmotor | Absence | 6/19 | 32% |
| Focal Seizure |  | 13/19 | 68% |
|  | Without Impairment of Consciousness | 5/19 | 26% |
|  | With Impairment of Consciousness | 8/19 | 42% |
|  | Evolving to Bilateral Convulsive | 3/19 | 16% |
| Unclassified Seizure |  | 5/19 | 26% |

Legend: Seizure counts and prevalence in participants within cohort who have an epilepsy diagnosis (n=19). Participants may have had multiple seizure types so percentages do not add up to totals.

**Supplemental Table 3**. Age of onset of seizures for participants with an epilepsy diagnosis, for those with this data available.

| Seizure Type |  | Age of Onset (Years)  (Mean, SD) | N available |
| --- | --- | --- | --- |
| Generalized (earliest) |  | 6.82 (4.9) | 11/12 |
| Motor subtypes | Tonic-Clonic | 7.67 (5.4) | 7/8 |
|  | Myoclonic | . | 0/0 |
|  | Clonic | 8.32 (6.5) | 2/2 |
|  | Tonic | 6.74 (2.7) | 3/4 |
|  | Atonic | 8.2 (0.9) | 2/2 |
|  | Epileptic Spasm | 3.73 (.) | 1/1 |
| Nonmotor | Absence | 6.04 (2.4) | 5/6 |
| Focal (earliest) |  | 10.64 (4.9) | 12/13 |
|  | Without Impairment of Consciousness | 6.25 (3.3) | 2/5 |
|  | With Impairment of Consciousness | 10.24 (5.3) | 7/8 |
|  | Evolving to Bilateral Convulsive | 9.60 (4.3) | 3/3 |

Legend: Mean and standard deviation of age of seizure onset (years). The number of participants with available dates to calculate onset age are in the last column. Participants may have had multiple seizure types so subgroup sample size does not add up to overall totals.
