# Supplementary figures and images for: Clinical, genetic, and cognitive correlates of seizure occurrences in Phelan-McDermid syndrome
Source: J Neurodev Disord. 2024 May 10;16:25. doi: 10.1186/s11689-024-09541-0 (PMC11084001; doi:10.1186/s11689-024-09541-0)

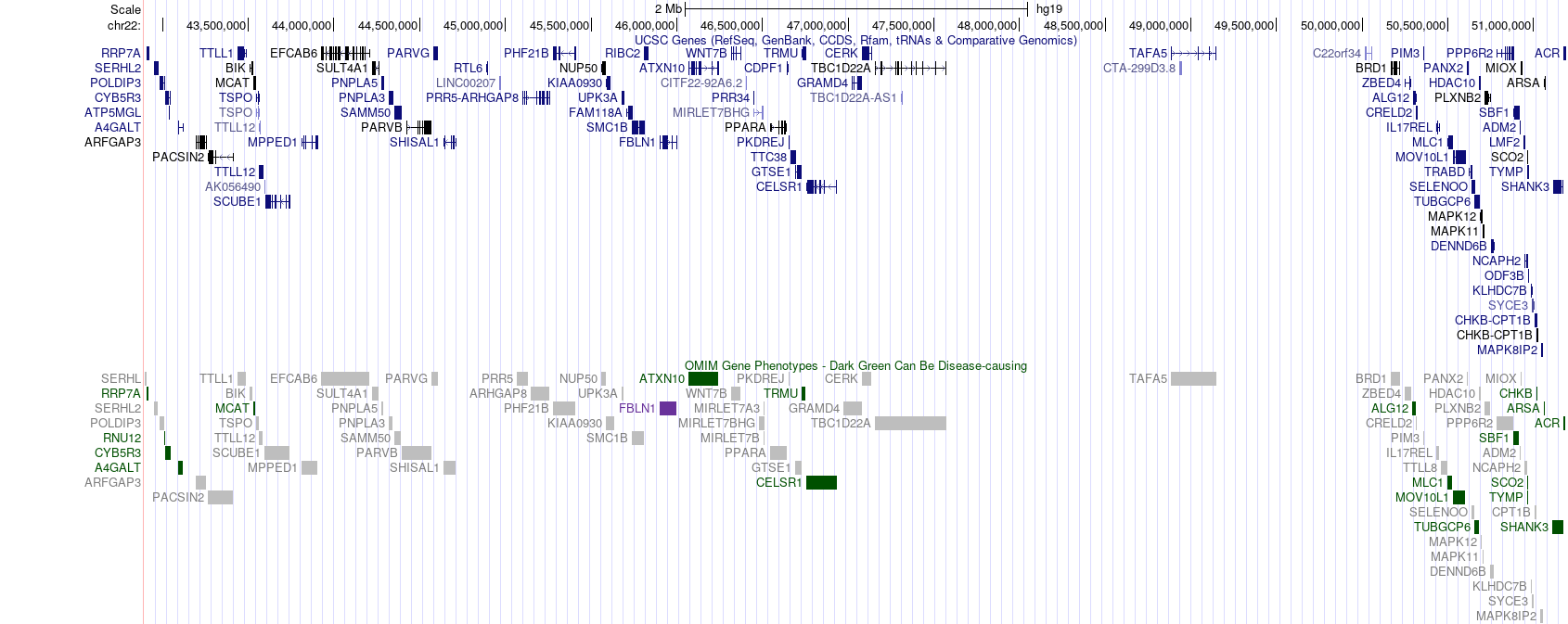

Supplement: Supplementary file 3 — Supplementary Material 3. [file 11689_2024_9541_MOESM3_ESM.png]
